# Using Relational Agents to Promote Exercise and Sun Protection: Assessment of Participants’ Experiences With Two Interventions

**DOI:** 10.2196/jmir.7640

**Published:** 2018-02-07

**Authors:** Marie A Sillice, Patricia J Morokoff, Ginette Ferszt, Timothy Bickmore, Beth C Bock, Ryan Lantini, Wayne F Velicer

**Affiliations:** ^1^ Department of of Psychiatry and Human Behavior Butler Hospital Brown University Providence, RI United States; ^2^ College of Arts and Sciences University of Rhode Island Kingston, RI United States; ^3^ College of Nursing University of Rhode Island Kingston, RI United States; ^4^ College of Computer and Information Science Northeastern University Boston, MA United States; ^5^ Department of of Psychiatry and Human Behavior The Miriam Hospital Brown University Providence, RI United States; ^6^ The Miriam Hospital Providence, RI United States; ^7^ Cancer Prevention Research Center Department of Psychology University of Rhode Island Kingston, RI United States

**Keywords:** relational agents, eHealth, exercise, sun protection, qualitative methods

## Abstract

**Background:**

Relational agents (RAs) are electronic computational figures designed to engage participants in the change process. A recent study, Project RAISE, tested the effectiveness of RAs, combined with existing computer-based interventions to increase regular exercise and sun protection behaviors. Results showed these interventions can be effective but need further development.

**Objective:**

The purpose of this study was to examine participants’ experiences using RAs to increase participant engagement and promote behavior change *.*

**Methods:**

A qualitative approach was primarily utilized. A 25-question interview guide assessed different components of participants’ experiences with the intervention, including motivation, engagement, satisfaction or dissatisfaction, quality of their interaction with the RA, and behavior change. Quantitative assessment of satisfaction was based on a scale of 1 to 10, with 1 representing least satisfied and 10 representing most satisfied. A summative analytic approach was used to assess individuals’ qualitative responses. A single analysis of variance (ANOVA) examined levels of satisfaction by gender.

**Results:**

Of the original 1354 participants enrolled in Project RAISE, 490 of 1354 (36%) were assigned to the RA group. A sample of 216 out of 490 (44%) participants assigned to the RA group completed the interventions, and follow-up assessments were contacted to participate in the semistructured interview. A total of 34 out of 216 (16%) completed the interview. Participants were motivated by, and satisfied with, the intervention. Participants viewed the RA as supportive, informative, caring, and reported positive behavior change in both exercise and sun protection. Some participants (15/34, 44%) noted the RA was less judgmental and less “overbearing” compared with a human counselor; other participants (12/34, 35%) said that the interaction was sometimes repetitive or overly general. The majority of participants (22/34, 65%) viewed the RA as an important contributor to their behavior change for exercise, sun protection, or both. Levels of satisfaction ranged between 7 and 10. There were no gender differences noted in levels of satisfaction (*P*=.51).

**Conclusions:**

RAs provide an innovative and attractive platform to increase exercise and sun protection behaviors and potentially other health behaviors.

## Introduction

### Background

During the last 30 years, numerous computer-based behavioral health interventions have been developed and have shown to be effective in clinical trials [1-4]. However, the effect sizes have been small due to a lack of engagement by a significant proportion of the population [3,4]. Relational agents (RAs) represent one of the recent innovative electronic communication technology approaches (electronic health) developed to increase engagement [5-9]. RA interventions for medication adherence and depression management have shown to be more efficacious in comparison with non-RA conditions [8,9]. A recent exercise and sun protection intervention tested the efficacy of RAs, combined with existing computer-based interventions, to increase participant engage and behavior change [9]. The study demonstrated that these interventions can be effective but need further development [9].

RAs are computational figures that incorporate information and communication technology to engage participants in the change process [5,7]. The RA uses speech, gaze, hand gesture, intonation, and other nonverbal modalities to emulate the experience of human face-to-face conversation with their users [5-9]. Specifically, the RA engages users through small talk, storytelling, humor, offering empathy, encouragement, and praise to help them to acquire and maintain the target behavior [6-9]. The interactions are in real time, allowing the user to ask the RA questions that may emerge during the session [6-9]. Furthermore, the RA is able to recall specific information, including trivial information, from a previous session to facilitate continuous engagement with the user. This feature of the RA provides the appearance of empathy and gives the impression that it (represented as he or she) is attentive to and thus cares about the user. This feature is vital to increasing engagement and building relationship overtime [6-9].

In-depth assessments of participants’ experiences with these interventions could potentially provide a better understanding of particular tenets that underlie the RAs’ interaction with participants that help to increase engagement and behavior change. Moreover, this knowledge can contribute to the improvement of specific functions or contents of these interventions that would further enhance participants’ interactions with the RAs. This study examined participants’ experiences with two longitudinal RA interventions for exercise and sun protection, titled Relational Agents Intervention for Sun Protection and Exercise (Project RAISE).

### Project RAISE

Insufficient exercise and prolonged unprotected exposure to ultraviolet light are associated with numerous adverse health effects including certain cancers [10-13]. National survey data show that a majority of American adults are not meeting the recommendations for regular exercise [12,13] and sun protection behaviors [10,11].

Project RAISE was a randomized controlled clinical trial comparing computer-tailored interventions (one for exercise and one for sun protection) with the following 3-group experimental design: (1) a multiple risk Internet group, (2) multiple risk Internet with RA condition, and (3) a control group [9]. The interventions were developed based on the transtheoretical model (TTM; [14,15]). Briefly, the TTM is a comprehensive framework with multiple dimensions of behavior and behavior change [16]. This model has been applied to various health behaviors [14-23], including exercise [16-18] and sun protection [21-23]. The core TTM constructs include decisional balance, stages of change, self-efficacy, and processes of change. These constructs work in concert throughout the process of behavior change [14,15].

An expert system intervention approach provided participants with unique matched information and intervention based on their health risks and attitudes toward exercise and sun protection [9]. A tracking chart helped participants monitor their exercise and sun protection behaviors weekly and over the course of the 12-month intervention. A workbook provided participants with activities designed to help them reduce physical inactivity, unprotected sun exposure, and progress to the next stage of change. Lastly, participants received a personalized email reminder by the RA if they did not access the program for 7 days. These reminders continued once a week until they accessed the intervention to help participants remain on track with the program [9].

Demographic information for race and gender was used to match participants to one of four RAs (see Multimedia Appendix 1 for pictures of the RAs). The RA maintained a conversational approach with participants throughout the program, while providing them with support and encouragement to engage in regular exercise and sun protection behaviors and eventually maintenance. Specifically, the RA acknowledged participants’ struggles or difficulties, showed empathy, and provided them with tailored strategies to overcome reported barriers for exercise (eg, finding the best time in your schedule to exercise or putting your sneakers in front your bed as a reminder or encouragement to exercise) and sun protection (eg, setting an alarm as a reminder to apply sunscreen before sun exposure) [9]. Participants who reached their weekly goals for exercise (eg, 30 min or more of moderate intensity level of physical activity (PA); [9]) or sun protection (eg, applied sunscreen regularly during the week before sun exposure; [9]) were acknowledged or complimented (eg, You did an excellent job! You should be proud of yourself) and given additional strategies for continued engagement [9]. Participants who did not reach their goals also received encouragement and tailored strategies that addressed related barriers. Moreover, the RA used small talk and storytelling about day-to-day life events (eg, how a friend resolves a problem with a coworker) throughout the intervention to engage users [9]. These conversation modalities also included humor or daily jokes. Participants interacted with the RA throughout the 12-month intervention. As previously noted, all interactions were in real time, allowing participants to ask the RA questions and receiving immediate answers [9].

The 12-months data showed that the RA intervention increased participant engagement [9]. The RA group viewed an average number of 0.142 sessions each week compared with the non-RA conditions, which viewed an average of 0.048 sessions [9]. A slightly higher percentage of RA participants (16.6) met recommended guidelines for regular PA (ie, 150 min of moderate to vigorous level of PA each week [15]) compared with the multiple risk Internet condition (16%) and control (14%) [9].

## Methods

### Study Design and Participants

A standardized open-ended qualitative interview was used to assess participants’ experiences, including level of satisfaction with the interventions. The use of qualitative interviews facilitates a greater understanding of participants’ experiences, including feelings, emotions, and opinions when compared with only quantitative assessments [24-26]. The following research questions were used to assess participants’ experiences:

1. What was the experience of individuals who participated in the intervention?
2. What were their reasons for participating in the intervention?
3. What expectations did individuals have before participating in the interventions?
4. What was their reaction and interaction with the RA?
5. What promoted consistent and increased level of participation among individuals?
6. What was the level of trust individuals had with the information provided?
7. How did individuals’ participation in the program influence their future behavior?
8. What intervention component or components id participants attribute most to their exercise and sun protection behaviors?
9. Did participants’ level of satisfaction (or dissatisfaction) differ for gender and race based on a scale of 1 to 10, where 10 was the “most satisfied” and 1 the “least satisfied”?
10. Did participants’ stage of change play a role in their experience with the interventions?

A pragmatic orientation paradigm consisting of three tenets was used to explore participants’ experiences with the different components of the intervention: prospective, prescriptive, and constructive [27,28]. From a health behavior perspective, the three tenets work in concert to enable the researcher to grasp a greater understanding of the individuals’ experiences by taking into account their expectations and assumptions regarding the approaches needed to help them engage and maintain the target behavior(s) [27]. Specifically, this approach assesses individuals’ experiences or their application of the prescribed strategies for achieving regular engagement in the target health behavior(s) [27,28]. The prospective and perspective tenets address participants’ levels of acceptability of the intervention design (eg, intervention content, length of intervention, delivery, and supportive function), their experience, and subsequent evaluation [27-30]. The constructive concept focuses on individuals’ experiences, as they are related to behavior change, as well as their recommended improvements of future and similar interventions [29,30].

Participants were recruited from across the United States from 2010 to 2012 for the original Project RAISE [9,19]. Eligible participants were in the in preaction stages (precontemplation, contemplation, or preparation) for exercise and sun protection. Additional inclusion criteria included aged 18 to 75 years, willingness to provide demographic information, ability to participate in PA, and Internet access. Participants were assessed on three occasions: baseline, 12 months (end of treatment), and 24 month follow-up (12 months after end of treatment) [9]. All of the intervention materials and assessments were administered over the Internet. Consent and other human subject protocols were approved by the University of Rhode Island Institutional Review Board, and research was conducted according to the American Psychological Association’s ethical guidelines. Participants completed surveys assessing key constructs of the TTM. The survey questions assessed behaviors relating to exercise, sun exposure, and behavior change constructs, including stage of change, decisional balance, self-efficacy, and processes of change [8].

A total of 1354 adults were enrolled in Project RAISE [9]. The RA condition consisted of 490 participants. Of that sample, 216 participants who completed the intervention, the 12-month and/or 24-month follow-ups were contacted over a 3-month period via telephone and/or mail to participate in this study.

### Measures

Demographic data collected in the Project RAISE study included information pertaining to gender, age, race, and stages of change for both exercise and sun protection. Stages of change assessed an individual’s level of readiness to engage in PA and sun protection behaviors. The stages were precontemplation (participants who were not consistently engaging in PA or protecting themselves from the sun, along with not intending to begin within the next 12 months); contemplation (participants not consistently engaging in PA or protective behaviors yet were seriously considering doing so within the next 12 months); and preparation (participants who were not currently engaging PA or protection but planned to start within the next 30 days). Satisfaction was assessed using a scale of 1 to 10, with 1 representing least satisfied and 10 representing most satisfied [9].

### Interview Guide

The 25-question interview guide was developed based on several meetings with a team of researchers with expertise in health promotion, qualitative methods, clinical interviewing, and measurement development. Interview questions included “what” and “how” questions (instead of “why” questions) to help illicit in-depth information about participants’ experience with the interventions [25,26]. The wording of questions and their order of presentation were consistent across participants. The guide included follow-up questions and probes developed to facilitate accuracy of information, including points for clarification. Two other researchers independently reviewed the interview guide for clarity. The first author completed all of the interviews. The interviews ranged between 12 to 30 min.

### Analysis

**Figure 1:**
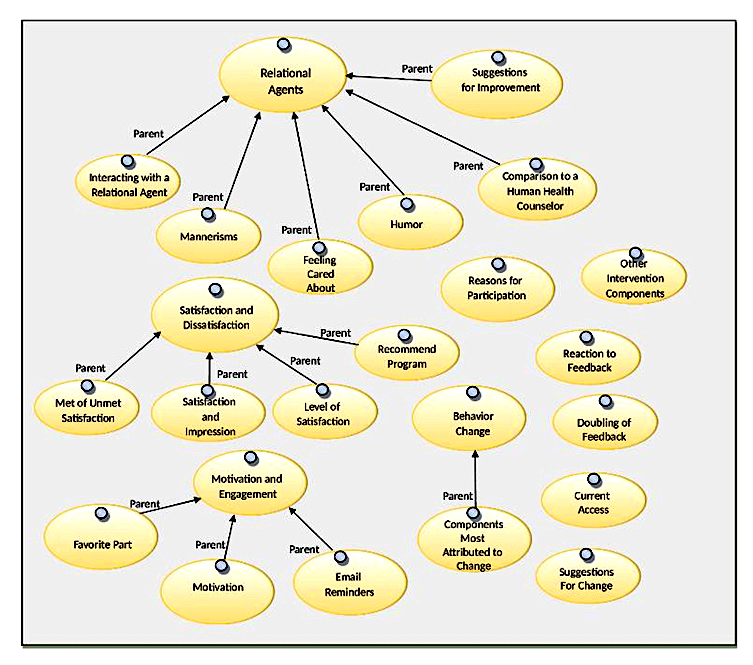
Analytic model.

All interviews were audio-recorded and were transcribed. Each transcribed interview was reviewed along with the original audio file to ensure an accurate transcription. Transcribed interviews contained participants’ ID number and were stored separately from demographic information. A preliminary coding analytic model was developed based on the interview guide over several meetings with the authors of this paper and was used to organize the data, using overarching categories, as well as different subcategories (see Figure 1).

The final coding was completed after reviewing the data and identifying emerging categories. Manifest content analysis was used to assess elements (and frequency of elements) that were present and countable in the data, such as particular words or wording [31,32]. Coding approaches consisted of a single word, phrases, complete sentences, and paragraphs in the larger sections of the interviews. Larger sections within the interviews were coded to provide context or clarification for use of single words or phrases. The NVivo 10 (QSR International) qualitative software was used for analyses [33]. Satisfaction was assessed using a scale of 1 to 10, with 1 representing least satisfied and 10 representing most satisfied. An analysis of variance (ANOVA) test was performed using Statistical Package for the Social Sciences (SPSS; IBM Corp) [34] and assessed potential gender differences for levels of satisfaction with the interventions.

### Methodological Rigor

Trustworthiness is a central component of qualitative research and consists of several strategies that ensure rigor and credibility of findings [31,32]. The strategies utilized to address trustworthiness in this study were prolonged engagement, member checking, reflexive journaling, double-coding, and triangulation [31,32]. For prolonged engagement, the first author conducted all of the interviews and was involved in data collection over four and a half months. 

The transcription of the interviews, as well as code development were completed, respectively, over 2 and 4 months. For member checking, the first author assessesed the accuracy of information by probing for additional information when necessary to enhance clarification. During the interview, the first author encouraged participants to share their true opinions or sentiments regarding their experirences with the intervention and suggestions for improving future and similar interventions. For reflexive journaling, the first author documented ideas and strategies in the development of codes, response categories, and subcategories. For dependability audit, the first author consulted with a qualitaitive expert, the third author, throughout the analytic process, giving special attention to all decisions made from this study’s inception to analyses and ultimate interpretation of results. For double coding, the third author randomly selected 20% of the interviews and coded them independently [31]. High consistency (100%) was noted between coders. Lastly, triangulation of quantitative and qualitative data was used to furrther assess participants’ experiences, as well as their levels of satisfaction with the interventions.

### Response Categories

The results of this study were determined by analyzing verbatim responses of participants. Responses were organized into 10 primary categories, assessing participants’ experiences with different components of the interventions. The categories were as follows: (1) reasons for participation, (2) motivation and engagement with the intervention, (3) satisfaction or dissatisfaction with the intervention, (4) RAs, (5) other intervention components, (6) behavior change, (7) the component(s) of intervention most attributed to change, (8) reaction to tailored feedback, (9) current access to the intervention, and (10) suggestions for change.

## Results

### Organization of Results

The number of participants who endorsed a category of responses is provided. Response exemplars are provided for most categories or subcategories along with the race and gender of the participant(s) (black male=BM, black female=BF, white male=WM, and white female=WF) and ID numbers.

### Participants

A total of 34 of 216 individuals who completed the RA interventions and/or 12- to 24-month follow-ups completed the semistructured interview. Participants were predominately white (31/34, 91%). For gender, there were 18 men (53%, 18/34) and 16 women (47%, 16/34). Participants were aged between age 20 and -75 years (mean=52.4, standard deviation=14.4). Nearly half (16/34, 47%) reported being married. Most of the participants were in the preparation stage (23/34, 68%) for exercise and in the precontemplation stage (26/34, 77%) for sun protection.

### Reasons for Participation

Responses fell into three primary categories: (1) interest in health improvement (13/34, 38%). One response example of this is *I needed to have a healthier lifestyle...* (656345, WF); (2) helping with research (11/34, 32%). One of the participants in this category noted, *I just figured I could provide some assistance with what they were doing maybe it might be able to help me in the future* (653768, WF); and (3) interest or curiosity about the program (6/34, 18%). A response exemplar for this category was *I just wanted to see what kind of program it was* (640486, WM).

### Motivation and Engagement

The questions used to explore motivation, engagement, and the specific components that promoted these behaviors were as follows:

1. What motivated you to keep using the program?
2. What was your favorite part of the program?
3. As a part of this program, you also received email reminders to access the program if you did not log on for a while. Did this help with keeping you on track?

Responses for question 1 resulted in eight categories of responses, with nearly half of the participants (16/34, 47%) recalling a desire to be healthy as their motivation. Other motivating factors reported were: accessibility of the program (3/34, 9%), interaction with the RA (2/34, 6%), email reminders (1/34, 3%), and completing the surveys (1/34, 3%).

For question 2, 13 of 34 participants (38%) noted the RA was their favorite part of the program. Two participants noted the following:

It [the RA] was so much easier and interactive.

652687, BF

...the fact that it pushed me to actually get up and move.

652207, WF

Twelve of the 34 participants (35%) did not recall having a favorite part of the program. Six of these participants (6/34, 18%) reported enjoying *all parts* of the program and/or found all the components to be equally helpful. Other participants repeated the same responses provided for reasons for participation (eg, helping with research).

For question 3, most participants recalled that the email reminders helped them to remain on track with the program (23/34, 68%). Fourteen participants (14/4, 41%) reportedly accessed the program *the same day or right after* receiving the email. Others mentioned accessing the program after 2 to 3 days (8/34, 24%). One of those participants mentioned the email reminder also served as a motivator to maintain regular exercise and sun protection behaviors, as indicated in the following quote:

It was great to have that reminder every couple of days to help me to think oh yeah, I’ve got to check my exercise...or how much exercise yesterday or how much today, or to put on sunscreen.

651780, WF

### Satisfaction or Dissatisfaction With the Intervention

Similar to the motivation and engagement category, satisfaction (and dissatisfaction) with the program were explored using several questions:

1. Would you say that the program met your expectations?
2. Describe your overall satisfaction or impression with the program?
3. On a scale of 1 to 10 where 10 is the most satisfied and 1 is the least satisfied, how would you rate your level of satisfaction with the program?
4. If a family member or friend were in a similar situation, would you recommend they participate in this program?

Additionally, for question 1, participants were asked to provide examples of how or reasons why the program met (or did not meet) their expectations. For question 1, 30 participants (30/34, 88%) reported that the program met their expectations. Two exemplars of responses are as follows:

...being informed about the dangers and using sunscreen and [information about] different types of exercise.

626637, WF

...[It] served as a reminder to exercise and protect my skin when I go outside.

615633, WM

For question 2, 31 participants (31/34, 31%) reported a positive impression with the program. Some exemplars of responses were as follows:

[I had] an amazing experience.

563466, WM

...it was very well thought out.

651780, WF

...it was well done.

652687, BF

Levels of satisfaction, based on a scale of 1 to 10, ranged from 7 to 10 (32/34, 94%). For question 4, 32 participants (32/34, 94%) mentioned recommending the program to either a family member or friend. Most of the participants explained that the program would help others learn the importance of exercise and sun protection, as well as serve as a motivator or a partner to help them maintain these behaviors overtime.

### Relational Agents

Seven questions were used to assess participants’ interaction with RA, as well as the physical attributes of the RA *.*

#### Interacting With a Relational Agent

For question 1, participants were asked, “what was it like for you to interact with a relational agent?” Most of the participants (32/34, 94%) described a positive interaction, and six of those descriptions were: *user-friendly* (587027, MW), *entertaining* (615633, WM), *interactive* (652440, WM), *easy and effortless* (652207, WF), *informative* (626033, WF), and that *[the RA]...made me feel better about myself* (651438; WM). Moreover, one participant described the RA as a *virtual pal* (627122, WF).

Two participants reportedly disliked the RA or their interactions with the RA (2/34, 6%). One participant mentioned that he would have preferred the *...interaction with a human being* (655948, WM). They were asked about their reasons for preferring a human being; they provided no additional information and reiterated that it was their preference.

#### Mannerisms

Participants were asked whether they felt the mannerisms of the RA were similar to those of a human being. (Did you feel her mannerisms, eg, facial features or hand gestures, were similar to those of a human being?). A total of 30 participants recalled the RA’s mannerisms were similar to those of a human being (30/34, 88%). The following response exemplars described 3 participants’ assessments of the RA’s mannerisms:

...he was enthusiastic, I guess in an upbeat way, very friendly.

587027, WM

I thought so. The way she moved her hands—that always seemed perfectly normal. I thought that the voice was cheery and professional.

651780, WF

Yes, definitely they were. The gestures were definitely believable.

652687, BF

#### Humor

Participants were asked whether they found the jokes that the RA shared with them throughout the course of the intervention to be humorous (During the program, the virtual health coach, or the RA shared many jokes with you. I was wondering if you found them to be humorous) *.* Eleven participants (11/34, 32%) recalled that the jokes were humorous, 8 participants (8/34, 24%) recalled that *some of the jokes* were humorous, and 13 participants (13/34, 38%) could not recall whether or not the jokes were humorous.

#### Comparison With Human Health Counselor

When comparing the RA with a human health counselor (how would you compare him or her with a human health counselor, eg, a personal trainer and/or a nurse?), 15 participants (15/34, 44%) mentioned the RA was *informative* and motivated them to maintain regular exercise and sun protection behaviors. Of that sample, 3 participants mentioned preferring the RA to a human health counselor or a personal trainer, as illustrated in the following quotes:

It [the RA] was less judgmental and less intimidating.

651780, WF

She reminds you enough to get the point across without being overbearing.

563466, WM

I have probably listened to her more that I would have an actual person.

653244, WF

In contrast, 12 participants (12/34, 35%) reported that their interaction with the RA was *limited* and explained that responses to their questions were at times *too general* or that certain responses became *repetitive* over the course of the intervention. A total of 7 participants (7/34, 21%) rejected the assumption that the RA could be compared with a human health counselor and thus, a real person, as indicated in the following quotes:

I can’t really make that comparison.

654855, WM

...it is not human.

657225, WM

#### Feeling Cared About

Participants were asked whether they felt the RA cared about them (Throughout your interaction with the RA, did you feel that she [or he] really cared about you?). In the first four interviews, the participants felt that the word *care* or *caring* is a human characteristic that cannot be attributed to either the RA and/or their interaction with the RA. This question was revised after these interviews. For the remaining interviews, participants were asked, “Throughout your interaction with the RA, did you feel that she (or he) really cared about your exercise and sun protection behaviors?” Most of the participants reportedly felt the RA cared about their engagement and maintenance of both exercise and sun protection (28/34, 82%). Some of the participants reported the following reasons:

If I am not mistaken, he addressed me by name.

615633, WM

It’s like having virtual pal online and being able to converse with them, and the virtual pal...care[s] how you are doing and that kind of thing.

651689, WF

Just the way she talked and the way she presented herself in the telling the benefits of sun protection and exercise.

656345, WF

#### Interaction With the Relational Agent Over Time

Participants were asked whether their interaction with the RA changed throughout the intervention. A total of 26 participants (26/34, 77%) recalled their positive feelings toward the RA remained the same throughout the intervention, 6 other participants (6/34, 18%) mentioned their feelings improved over time as they became more familiar with the RA and/or with the routine of the program, and one participant noted the RA became *like a friend*:

I mean he obviously became more familiar as I interacted more and more. [I] got more use to what to expect out of it so I think, like, an acquaintance becoming a friend as you sort of interact with them more and more.

586627, WM

#### Suggestions for Improvement

Participants were asked whether they would change anything about the RA. A total of 20 participants (20/34, 59%) mentioned the RA needed no improvements. An exemplar of those responses is as follows:

No, I think she did a wonderful job. If I wanted somebody more real or something like that, I am sure you can get a video of a real person doing that. This was better to me.

563466, WM

A total of 12 participants (12/34; 35%) provided various suggestions for improvement, including improving the *monotone* voice of the RA to a normal pitch (4/34, 12%) and allowing the user to choose an RA of the opposite gender, similar race, and age groups (3/34, 9%).

### Other Intervention Components

Participants were asked whether there were other things they liked about the program. A total of 14 participants (14/34, 41%) liked the information on the health benefits of exercise and sun protection. Other participants mentioned the program and/or the RA served as a constant *motivator* to become and remaining healthy (14/34, 41%). Moreover, 4 other participants (4/34, 12%) recalled that they liked the *ease* of the program. Two of those participants stated the following:

...it really was easy to use.

653244, WM

I could go at own pace.

615633, WM

### Behavior Change

Participants were asked about the ways in which the program helped them change their exercise and sun protection behaviors (How has the program been helpful in changing your exercise and sun protection behaviors?). A total of 11 participants (11/34, 32%) mentioned that the program helped them to engage in regular exercise. Two response exemplars are as follows:

The activity part was really helpful. If I can park a little further in the parking lot and walk. I have taken a lot to heart.

639021, WM

It pushed me to actually get up and move.

652207, WF

A total of 8 participants (8/34, 24%) noted that the program helped them to engage and maintain sun regular protection behaviors. Two exemplars of responses are as follows:

Before I started doing that I really didn’t use sunscreen at all. I started using sunscreen...more than I thought I would ever use.

653244, WF

I started using sunscreen. I actually use [SPF] 70 proof. I use hats more than I did before. I’ve stayed in the shade...and put more umbrellas in our backyard so we have shading that we need.

656345, WF

A total of 7 participants (7/34, 21%) reported a change in both exercise and sun protection. Two exemplars of responses are as follows:

It served as a reminder to exercise and protect my skin before going outside.

615633, WM

...[I apply] sunscreen and other protective things. I exercise.

656345, WF

Four other participants (4/34, 12%) stated the program served as reinforcement for continuing with their own routines for exercise. Because eligible participants were not currently exercising regularly at baseline, it is possible they were referring to exercise they engaged in irregularly, or to a routine they had engaged in at some time before enrolling in the study. Two participants (2/34, 6%) reported being *more aware* about the importance of sun protection behaviors as preventions against skin cancer. Lastly, two other participants (2/34, 6%) stated their participation in the program did not lead to change in either exercise or sun protection behaviors. They provided no additional information for the lack of behavior change.

### Components Most Attributed to Change or Awareness

On the basis of participants’ responses to the behavior change category, they were asked about the component(s) of the intervention they attributed most to their behavior change (n=26), becoming *more aware* of the benefits of sun protection and reducing cancer risk (n=2), or that served as a reinforcement for their own exercise routines (n=4; Was it the virtual health coach, which was the RA; the reports; the workbook; or the tracking charts?). A total of 22 participants (22/26, 85%) attributed their behavior change primarily to the RA, and 10 participants (10/26, 38%) noted that the combination *of all* aforementioned components was equally helpful in changing their exercise and/or sun protection behaviors.

### Reaction to Tailored Feedback

Participants were asked if the tailored feedback they received throughout the intervention helped to change their exercise and sun protection behaviors (As a part of the program, you received some feedback about ways to increase engagement regular PA and sun protection. For instance, you may have heard pr read that support from others is extremely helpful to meet your healthy lifestyle goals. As a result, you may have begun working out with a friend to help achieve your goals) *.* A total of 29 participants (29/34, 85%) reported that the feedback helped them to engage in and maintain regular exercise and sun protection behaviors; 3 participants (3/34, 9%) reported that some of the feedback messages for exercise were irrelevant for their lifestyles. Two response exemplars are as follows:

It kind did not apply to me because I had an exercise regimen and with my schedule, it’s hard to connect with somebody.

586372, BM

I live in a village of 196 people, and we’ve got 63 acres in back of us. No, I can’t ask someone to [go] out [and walk] with me.

651005BM

### Doubting of Feedback

All participants were asked whether they ever doubted any of the feedback or statements that were given to them. All 34 participants (100%) responded “no” to this question. A response exemplar is as follows:

No. From having done my own research prior to this, research on sun exposure and exercise and things like that, I found that everything that you guys said was very believable.

563466, WM

### Current Access

Participants were asked whether they would have preferred current or continued access to the program. A total of 18 participants (18/34, 53%) reported “yes” to current access. Of that sample, 6 participants mentioned the program would continue to serve as a motivator or as a *good prod* (n=2) for maintaining regular exercise and sun protection behaviors or serve as a reference for *reviews* or suggestions for exercise and sun protection (n=4). Two exemplars of responses are as follows:

I guess it would be again, a good prod cause sometimes I kind of forget about doing things I wanted to do and get bogged down in different priorities.

586372, BM

Yes...just having motivation...you get on there and you can go through the saver tools of answering the questions about how much you are exercising, and making sure that you have sunscreen on and wearing the proper gear.

651780, WF

Conversely, 12 participants said that they did not want access to the program. Most of those participants stated they enjoyed the program and learned a lot from it; however, they felt that continuing access would not lead to any additional impact on their behaviors. Four other participants were reportedly *unsure* to whether they would like current access to the program.

### Suggestions for Improvement

Participants were asked to report their suggestions for improving the overall program (I was wondering if there is anything that you like us to do differently? Are there some things you would have liked to see more or less of ?). A total of 6 participants (6/34, 18%) mentioned that they would not make any changes to the program; 22 participants (22/34, 65%) repeated the same responses they provided in the Suggestions for Change subcategory of the RA category. Three of the 6 participants who provided suggestions for improvement shared the following:

Perhaps at some point through the program have a meeting with an actual [human] health agent over the phone.

587027, WM

If you could actually put the program on your iPhone as well, or you could do it on the go.

651780, WF

...an option to shorten your time on there. I guess...sort of shut it off in the middle of whatever you were doing so you later finish it.

652440, MW

### Quantitative Assessments of Satisfaction

Potential gender differences in level of satisfaction were examined using an ANOVA test. Results that men and women did not significantly differ in levels of satisfaction: *F*_1,33_=0.399, *P*=.51; η_p_^2^ =.002.

## Discussion

### Principal Findings

This study explored the experience of 34 individuals who completed two RA interventions for exercise and sun protection, using primarily a qualitative approach. The primary purpose of this study was to explore participants’ reaction to and interaction with the RA and determine the importance of this component in promoting exercise and sun protection behaviors in this sample. Moreover, this study investigated participants’ experience with all components of the program, as well as their reasons for participating in an exercise and sun a protection intervention. Reasons for participation have shown to be associated with motivation and engagement overtime. Furthermore, individuals’ expectations of a program for exercise and sun protection and their proximity to their actual experience subsequently influenced their evaluation.

Participants’ reasons for participation, which included health improvement, coincided with factors for motivation and sustained engagement in the program. Approximately, 13 participants (13/34, 38%) noted the RA as their “favorite,” and 6 participants (6/34, 18%) reportedly liked and or enjoyed all parts of the program, including the RA. A number of participants did not express having a favorite part of the program. The wording of this question, “what was your favorite part of the program,” assumes that every participant had a favorite part of the program. However, it is unclear whether an alternative question such as “did you have a favorite part of the program?” would have produced different results.

The personalized email reminder from the RA helped most participants (23/34, 68%) access the program regularly and for some participants served as a reminder and motivator to exercise and protecting their skin from the sun if they had not been doing so regularly in the last few days. These findings indicate that periodic reminders are an important component to promote consistent engagement in health intervention, as well as helping individuals to think about their exercise and sun protection behaviors.

A total of 30 participants (30/34, 88%) reported being satisfied with the program. Satisfaction, in general, was noted as an outcome of met expectations and positive impression with the program. Most participants described the program as “informative,” “motivational,” and “accessible.” Levels of satisfaction ranged between 7 and 10. Most participants (26/34, 77%) reported behavior change in exercise and/or sun protection. Stage of change for exercise and sun protection did not appear to be associated with participants’ experiences and/or their evaluations of the interventions. The RA was the intervention component that attributed most to behavior change. Thus, the RA was shown to be an important platform for disseminating health intervention, increasing behavior change and thus, a valuable tool for health promotion research.

Regarding behavior change, participants were asked “how has the program been helpful in changing your exercise and sun protection behaviors?” This wording assumes that all participants adopted exercise and sun protection behaviors as a result of their participation in Project RAISE. Some participants may have felt compelled to an untruthful report of their behavior change. However, socially desirable responses are less likely when the interview is conducted over the telephone, and the topic is noncontroversial [35,36]. The interviewer also emphasized to participants the importance of reporting their true experience with and/or sentiments about the program to help improve future and similar interventions to minimize incorrect reporting.

Participants’ responses to feedback and or recommendations for engagement in exercise and sun protection indicate that they found them, overall, to be credible and/or effective. However, 3 participants mentioned that some of the recommendations for exercise were irrelevant to their lifestyles, which could be attributed to the wording of this question. As part of this question, participants were given the example of tailored feedback for exercise they may have received during the program: “For instance, you may have heard or read that support from others is extremely helpful to meet your healthy lifestyle goals. As a result, you may have begun working out with a friend to help achieve your goals. Did you find the suggestions helpful?” It is possible their responses were based solely on the singular message for exercise. This question is somewhat leading and may have influenced their responses. In hindsight, this question should have served only as a potential probe to participants who had difficulty answering or remembering messages or feedback for exercise. Additionally, two separate questions should have been used to assess tailored feedback messages or exercise and sun protection.

A little over 50% of participants wanted continued access to the intervention materials beyond the 12-month period. It may be helpful if future studies include a website on information or suggestions for exercise for 6 months after the intervention. However, this option might be costly and thus impractical for most health programs. Another option is to allow participants to save or print information, including feedback and recommendations, throughout the intervention.

A few participants made suggestions for improving future and similar programs. These included (1) disseminating the program to mobile phone, (2) allowing users to stop anytime during a session and to continue from that section later, and (3) using shorter surveys. A mobile phone–delivered RA intervention would increase dissemination by enabling participants to access the program anywhere and at their convenience. The ability for participants to stop at a section of a session and continue from there a later time would provide participants with more flexibility to complete the program. Moreover, the use of shorter surveys might prevent participants from feeling overburdened. However, the shortening of surveys could impact their psychometrics, hinder data collection, and thus, tailoring.

### Strengths and Limitations

This study has several strengths. To our knowledge, this is the first study to conduct an in-depth assessment of participants’ experiences with RA interventions. Moreover, this study assessed the role of the RA in increasing participation engagement, as well as potential behavior change. This study also demonstrated the role of a primarily qualitative approach in developing a greater level of understanding of participants’ experiences with RA interventions for exercise and sun protection. However, several limitations should be noted. First, the interview was conducted a year after participants completed the 12-month intervention. This may have impacted some participants’ ability to recall experiences of certain components (eg, jokes shared by RA) and their evaluation. While the RA intervention was distinctive and individuals may be more likely to remember it compared with a standard or non-RA intervention, the completion of interviews soon after the 12-month program could have provided more details on participants’ experiences. Second, the sample consisted of individuals who completed the 12-month intervention. Thus, the study results may not be representative of most participants’ experiences with the intervention. It is likely that individuals who were dissatisfied with the intervention simply disregarded our invitation to complete the interview. Moreover, it is possible that nonresponders had personal or professional priorities during that period that prevented them from participating in this study. Third, there are inherent limitations of the qualitative analyses used in this study. Content analysis is subjective and can be influenced by researchers’ biases. The subjectivity of content analysis can lead to incorrect interpretation of data, as it is influenced by a researcher’s skills and idiosyncrasies. This study used numerous strategies recommended to ensure the credibility of data collection, analyses, and interpretations.

Lastly, the sample size of black participants was too small to conduct an in-depth assessment of this group’s experience with the intervention. Thus, it is unclear whether black participants would have reported similarly positive experiences with the RAs in comparison with their white counterparts. Moreover, the small sample size for this group prevented the assessment of gender in black participants’ experiences with the RAs. Future studies should assess the experience of individuals of different racial and ethnic groups with these interventions. Moreover, a large sample of men and women is needed to explore potential gender differences in experiences with these interventions.

### Finding Implications and Conclusion

This study makes substantial contributions to the understanding of participants’ experiences with RA interventions and the importance of this complementary component in increasing participant engagement and promoting behavior change. Participants viewed the RA as interactive, supportive, and motivational, which were found to be central factors in their behavior change for exercise and sun protection. These findings suggest that the RA could potentially promote engagement in other health behaviors. Moreover, because these RA interventions are easy to use and participants can access them at their own convenience, participation burden is less compared with traditional clinical trials that require numerous face-to-face sessions and over a long period. Moreover, RA interventions require less clinical staff and no lab space to implement and thus, might be a more cost-effective approach to promoting health behavior change. Future studies should conduct cost analysis of RA interventions compared with standard behavioral health interventions.

## Appendix

Multimedia Appendix 1 Project RAISE relational agents.

## Abbreviations

BF: black female
BM: black male
RAISE: Relational Agent Intervention for Sun Protection and Exercise
RA: relational agent
TTM: transtheoretical model
WF: white female
WM: white male

## References

[1] Adams RJ (2010). Improving health outcomes with better patient understanding and education. Risk Manag Healthc Policy.

[2] McDermott L, Yardley L, Little P, Ashworth M, Gulliford M, eCRT Research Team (2010). Developing a computer delivered, theory based intervention for guideline implementation in general practice. BMC Fam Pract.

[3] Krebs P, Prochaska JO, Rossi JS (2010). A meta-analysis of computer-tailored interventions for health behavior change. Prev Med.

[4] Lustria ML, Cortese J, Noar SM, Glueckauf RL (2009). Computer-tailored health interventions delivered over the Web: review and analysis of key components. Patient Educ Couns.

[5] Bickmore TW, Caruso L, Clough-Gorr K, Heeren T (2005). 'It's just like you talk to a friend' relational agents for older adults. Interact Comput.

[6] Battaglino C, Bickmore T (2015). Association for the Advancement of Artificial Intelligence.

[7] Bickmore TW, Silliman RA, Nelson K, Cheng DM, Winter M, Henault L, Paasche-Orlow MK (2013). A randomized controlled trial of an automated exercise coach for older adults. J Am Geriatr Soc.

[8] Campbell RH, Grimshaw MN, Green GM (2009). Relational agents: a critical review. Open Virtual Real J.

[9] Velicer W, Redding C, Blissmer B, Babbin S, Paiva A, Bickmore T, Johnson J (2014). Using relational agents to increase engagement in computer-based interventions: preliminary outcomes. Int J Behav Med.

[10] Centers for Disease ControlPrevention (CDC) (2012). Sunburn and sun protective behaviors among adults aged 18-29 years--United States, 2000-2010. MMWR Morb Mortal Wkly Rep.

[11] (2014). U.S. Department of Health and Human Services, Office of the Surgeon General.

[12] U.S. Cancer Statistics Working Group (2016). Nccd.cdc.

[13] U.S. Department of Health and Human Services (2015). Surgeongeneral.

[14] Velicer WF, Prochaska JO, Fava JL, Rossi JS, Redding CA, Laforge RG, Robbins ML (2000). Using the transtheoretical model for population-based approaches to health promotion and disease prevention. Homeost Health Dis.

[15] Prochaska JO, Velicer WF (1997). The transtheoretical model of health behavior change. Am J Health Promot.

[16] Hall KL, Rossi JS (2008). Meta-analytic examination of the strong and weak principles across 48 health behaviors. Prev Med.

[17] Scioli-Salter E, Sillice M, Mitchell K, Rasmusson A, Allsup K, Biller H, Rossi J (2014). Predictors of long-term exercise maintenance among college aged adults: role of body image anxiety. Calif J Health Promot 2014 May.

[18] Blissmer B, McAuley E (2002). Testing the requirements of stages of physical activity among adults: the comparative effectiveness of stage-matched, mismatched, standard care, and control interventions. Ann Behav Med.

[19] Sillice MA, Babbin SF, Redding CA, Rossi JS, Paiva AL, Velicer WF (2018). Psychometric assessment of the processes of change scale for sun protection. Psychol Health Med.

[20] Sillice MA, Babbin SF, Paiva AL, Redding AC, Rossi JS, Velicer WF (2017). Assessing demographic differences in decisional balance for smoking prevention and temptations to try smoking among adolescent subgroups. Tob Prev Cessat.

[21] Babbin SF, Yin H, Rossi JS, Redding CA, Paiva AL, Velicer WF (2015). Reducing sun exposure for prevention of skin cancers: factorial invariance and reliability of the self-efficacy scale for sun protection. J Skin Cancer.

[22] Yin HQ, Rossi JS, Redding CA, Paiva AL, Babbin SF, Velicer WF (2014). Validity and stability of the decisional balance for sun protection inventory. J Skin Cancer.

[23] Prochaska JO, Velicer WF, Redding C, Rossi JS, Goldstein M, DePue J, Greene GW, Rossi SR, Sun X, Fava JL, Laforge R, Rakowski W, Plummer BA (2005). Stage-based expert systems to guide a population of primary care patients to quit smoking, eat healthier, prevent skin cancer, and receive regular mammograms. Prev Med.

[24] Patton MQ (1980). Qualitative Evaluation Methods.

[25] Seidman I (2013). Interviewing as Qualitative Research: A Guide for Researchers in Education and the Social Sciences. 3rd Edition.

[26] Kvale S, Brinkmann S (2009). Learning the Craft of Qualitative Research Interviewing, 2nd edition.

[27] Royse D, Thyer BA, Padgett DK (2015). Program Evaluation: An Introduction to an Evidence-Based Approach. 6th Edition.

[28] Rescher N (1999). Realistic Pragmatism: An Introduction to Pragmatic Philosophy.

[29] Bohman J (2002). How to make a social science practical: pragmatism, critical social science and multiperspectival theory. Millennium.

[30] Cronin JJ, Brady MK, Hult GM (2000). Assessing the effects of quality, value, and customer satisfaction on consumer behavioral intentions in service environments. J Retailing.

[31] Hsieh HF, Shannon SE (2005). Three approaches to qualitative content analysis. Qual Health Res.

[32] Miles MB, Huberman AM, Saldaña J (2013). Qualitative Data Analysis: A Methods Sourcebook. 3rd Edition.

[33] (2012). QSR International Pty Ltd.

[34] (2012). IBM Corp.

[35] van de Mortel TF (2008). Faking it: social desirability response bias in self-report research. Aust J Adv Nurs.

[36] Collins K, Nicolson P (2002). The meaning of 'satisfaction' for people with dermatological problems: reassessing approaches to qualitative health psychology research. J Health Psychol.

