# Supplementary figures and images for: Using Relational Agents to Promote Exercise and Sun Protection: Assessment of Participants’ Experiences With Two Interventions
Source: J Med Internet Res. 2018 Feb 7;20(2):e48. doi: 10.2196/jmir.7640 (PMC5822036; doi:10.2196/jmir.7640)

## Multimedia Appendix 1

### Relational Agents In Project RAISE

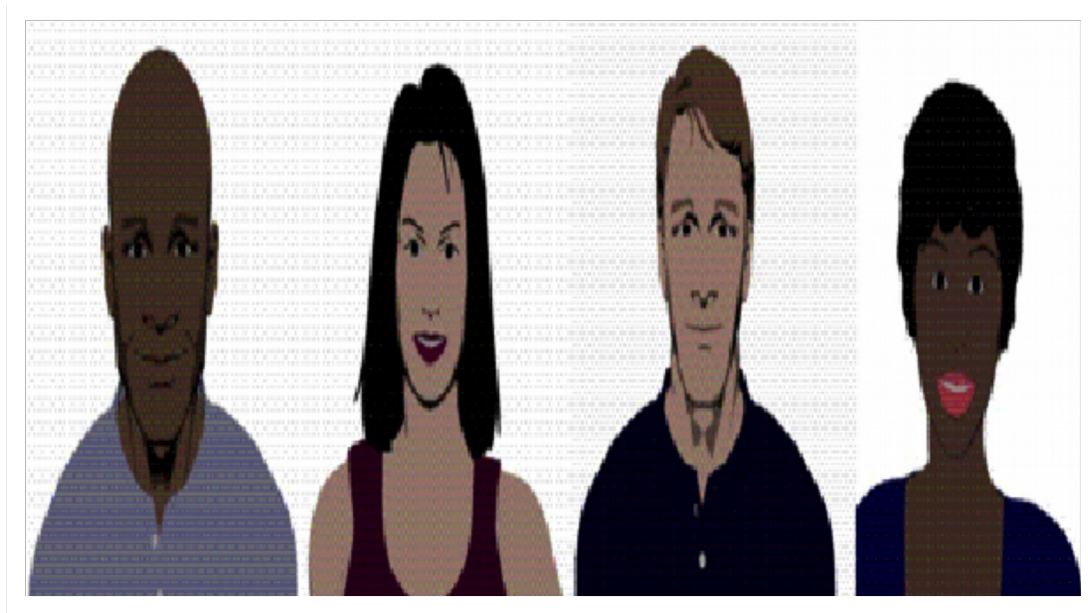

Supplement: Multimedia Appendix 1 [file jmir_v20i2e48_app1.pdf]
